# Does country of resettlement influence the risk of suicide in refugees? A case-control study in Sweden and Norway

**DOI:** 10.1017/S2045796021000512

**Published:** 2021-10-11

**Authors:** R. Amin, E. Mittendorfer-Rutz, L. Mehlum, B. Runeson, M. Helgesson, P. Tinghög, E. Björkenstam, E. A. Holmes, P. Qin

**Affiliations:** 1 Division of Insurance Medicine, Department of Clinical Neuroscience, Karolinska Institutet, SE-171 77 Stockholm, Sweden; 2 National Centre for Suicide Research and Prevention, Institute of Clinical Medicine, University of Oslo, Sognsvannsveien 21, 0374 Oslo, Norway; 3 Centre for Psychiatry Research, Department of Clinical Neuroscience, Karolinska Institutet, Stockholm County Council, 112 81 Stockholm, Sweden; 4 Swedish Red Cross University College, Hälsovägen 11, 141 57 Huddinge, Sweden; 5 Division of Psychology, Department of Clinical Neuroscience, Karolinska Institutet, SE-171 77 Stockholm, Sweden; 6 Department of Psychology, Uppsala University, Von Kraemers allé 1A and 1C, SE-752 37 Uppsala, Sweden

**Keywords:** case-control studies, country of birth, duration of residence, labour market marginalisation, migration, refugees, suicide

## Abstract

**Aims:**

Little is known regarding how the risk of suicide in refugees relates to their host country. Specifically, to what extent inter-country differences in structural factors between the host countries may explain the association between refugee status and subsequent suicide is lacking in previous literature. We aimed to investigate (1) the risk of suicide in refugees resident in Sweden and Norway, in general, and according to their sex, age, region/country of birth and duration of residence, compared with the risk of suicide in the respective majority host population; (2) if factors related to socio-demographics, labour market marginalisation (LMM) and healthcare use might explain the risk of suicide in refugees differently in host countries.

**Methods:**

Using a nested case-control design, each case who died by suicide between the age of 18 and 64 years during 1998 and 2018 (17 572 and 9443 cases in Sweden and Norway, respectively) was matched with up to 20 controls from the general population, by sex and age. Multivariate-adjusted conditional logistic regression models yielding adjusted odds ratios (aORs) with 95% confidence intervals (95% CI) were used to test the association between refugee status and suicide. Separate models were controlled for factors related to socio-demographics, previous LMM and healthcare use. Analyses were also stratified by sex and age groups, by refugees' region/country of birth and duration of residence in the host country.

**Results:**

The aORs for suicide in refugees in Sweden and Norway were 0.5 (95% CI 0.5–0.6) and 0.3 (95% CI 0.3–0.4), compared with the Swedish-born and Norwegian-born individuals, respectively. Stratification by region/country of birth showed similar statistically significant lower odds for most refugee groups in both host countries except for refugees from Eritrea (aOR 1.0, 95% CI 0.7–1.6) in Sweden. The risk of suicide did not vary much across refugee groups by their duration of residence, sex and age except for younger refugees aged 18–24 who did not have a statistically significant relative difference in suicide risk than their respective host country peers. Factors related to socio-demographics, LMM and healthcare use had only a marginal influence on the studied associations in both countries.

**Conclusions:**

Refugees in Sweden and Norway had almost similar suicide mortality advantages compared with the Swedish-born and Norwegian-born population, respectively. These findings may suggest that resiliency and culture/religion-bound attitudes towards suicidal behaviour in refugees could be more influential for their suicide risk after resettlement than other post-migration environmental and structural factors in the host country.

## Introduction

As global forced displacement has been one of the major public health issues of our time, Europe has seen a marked increase in the number of refugees since 2000 (UNHCR, 2020). The Scandinavian countries have also received an unprecedented high number of refugees in the last decades. During 2004–2019, Sweden and Norway granted residence to around 337 000 and 103 000 refugees, respectively (Statistics Norway, 2021; Statistics Sweden, 2021). In 2019, persons with a refugee background comprised 6.5 and 4.4% of the total population of Sweden and Norway, respectively.

For refugees, migration poses particular difficulties, e.g. war, torture and persecution in the pre-migration phase, peri-migratory perilous journeys, separation from or bereavement of family members and post-migration harsh circumstances in refugee camps (Tinghög, 2009). Exposure to stressful events, both during the early years and later in life, has well-documented effects on the development of mental ill-health and suicidal behaviour (Thordardottir *et al*., 2020). This has been shown in people who have been directly exposed to stressful war-related events with consequential increased rates of suicidal behaviour and mental ill-health in the following years after the war-stressor exposure (Karam *et al*., 2012). The association between these stressful events and suicidal behaviour could possibly be explained by the effect such trauma exposure may have on increases in the risk of mental disorders, which in turn increases the risk of suicidal behaviour. However, it could also be explained by the aggravating effect on the sense of distress that people with pre-existing mental disorders may experience (Karam *et al*., 2012). Despite this, few studies investigated the risk of suicide in refugees.

A study reported a significantly lower risk of suicide for only male refugees in Denmark compared with the Danish-born (Norredam *et al*., 2013). Hollander *et al*. found a lower risk of suicide in refugees, compared with both the non-refugee migrants and the host population in Sweden (Hollander *et al*., 2020). Two recent cohort studies also reported lower risks of suicide in refugees, by country of birth, than the host population in Sweden (Bjorkenstam *et al*., 2020; Amin *et al*., 2021*a*). Previously, better health outcomes in migrants were proposed to be partly explained by the so-called ‘healthy migrant effect’, which implies a selection effect, assuming that migrants have better health than the population in their birth country (Norredam *et al*., 2013).

Several factors on the individual and structural level have been shown to contribute to the multifactorial aetiology of suicide (Hawton and van Heeringen, 2009; Turecki, 2021). This is reflected in different models, such as the stress-diathesis model of suicidal behaviour which emphasises the interaction between hereditary susceptibility and stressful life events (van Heeringen, 2012). While refugees fleeing from the same region/country may have experienced such pre-migration stressful events in a similar way, the post-migration experiences may differ between host countries. Consequently, the effect of post-migration stress on mental ill-health and suicidal behaviour may vary between refugee groups from the same origin country resettling in different host countries. Therefore, the risk of suicide in refugees in host countries may depend on differences regarding several country-level factors. Healthcare system, social insurance and migration policies differ between countries which can differentially affect refugees' health and social situation in different host countries. Moreover, integration policies related to participation in the labour market can differ among host countries (Tinghög, 2009). Country-level structural factors, e.g. national healthcare and social insurance regulations, might shape health and social inequalities in refugees compared to the host population. Therefore, it is important to study if inter-country differences shaping post-migration experiences in different host countries may influence the suicide risk in refugees.

Comparing such inter-country differences in two Scandinavian countries, i.e. Sweden and Norway, offers several possibilities. While country-level suicide rates in these countries are comparable (WHO, 2021), and other similarities regarding governing and structural institution-building exist, there are also some notable differences. Previous studies reported that refugees in Sweden (Amin *et al*., 2021*b*), especially recently arrived refugees (Brendler-Lindqvist *et al*., 2014; Hollander *et al*., 2020), had lower specialised psychiatric healthcare use than refugees in Norway (Abebe *et al*., 2017), suggesting refugees in Sweden probably face more barriers to access healthcare. Moreover, social insurance regulations concerning labour market marginalisation (LMM) factors (unemployment, sickness absence benefits and disability pension) are also stricter in Sweden than in Norway (Helgesson *et al*., 2017; Norwegian Ministry of Labour and Social Affairs, 2020; Swedish Social Insurance Agency, 2020). During the last 10 years, annual unemployment rates in Norway have been much lower than in Sweden (Eurostat, 2021). Furthermore, due to a somewhat more generous immigration policy, Sweden has also received more refugees from Africa and Asia than Norway has in the past two decades (Statistics Norway, 2016; Swedish Migration Agency, 2020). Although integration policies for newly arrived refugees are rather similar in both countries (Hernes *et al*., 2019), labour market integration is somewhat better in Norway than in Sweden (Calmfors and Gassen, 2019).

Based on the similarities and differences mentioned above regarding country-level structural factors between Sweden and Norway, it might be hypothesised that the risk of suicide, in comparison with the respective host population, may be comparatively lower in refugees resettling in Norway than refugees in Sweden. On the other hand, if cultural factors from refugees' country of birth and their resiliency play a more vital role in the risk of suicide in refugees than structural factors in the new host country, then it is expected that this risk will not vary much between refugee groups in Sweden and Norway. Therefore, using data from Sweden and Norway for parallel analyses to investigate to what extent inter-country differences in structural factors, if any, explain the risk of suicide in refugees presents a unique opportunity in this field of research.

Regarding the association between refugee status and suicide, it is important to consider refugees' duration of residence following resettlement. Previously, the risk of suicide among migrants (all migrants including refugees) was reported to be converging with the suicide risk in the host population over time (Hollander *et al*., 2020). However, whether this pattern is also true for refugees is not yet investigated. Likewise, as suicide rates vary considerably by sex and age group (WHO, 2019), and there are generally higher proportions of younger men among refugees than the host population (Amin *et al*., 2021*a*), it is crucial to investigate if sex and age play any modifying role in these associations.

## Aims

We aimed to investigate (1) the risk of suicide in refugees resident in Sweden and Norway, in general, and according to their sex, age, region/country of birth and duration of residence, compared with the risk of suicide in the majority population in the respective host country; (2) if factors related to socio-demographics, LMM and healthcare use might explain the risk of suicide in refugees differently in host countries (Sweden and Norway).

## Materials and methods

### Design and study population

Using a nested case-control study design, all 17 572 and 9443 individuals aged 18–64 years who died by suicide during 1998–2018 in Sweden and Norway, respectively, were included as cases. The choice of this time period is based on the availability and comparability of data in both countries. For every individual who died by suicide, up to 20 controls were randomly selected from the general population who were alive and of the same sex and age as the case at the time of suicide. This method yielded 351 440 and 188 860 controls in Sweden and Norway, respectively.

### Data sources

For Sweden, individuals who died by suicide were identified from the Swedish Cause of Death register. Then, data linkages were done from the following registers (1) Statistics Sweden: sex, age, region and country of birth, educational level, family situation, type of residential area, number of annual net days with sickness absence benefit, disability pension and number of annual days with unemployment from the LISA database and reason for residence in Sweden (e.g. refugee status) from the STATIV database; (2) the National Board of Health and Welfare: date and cause of inpatient and specialised outpatient healthcare from the National Patient Register.

Cases in Norway were identified from the Norwegian Cause of Death register. This information was then linked to the following registers with individual data: (1) the Central Population Register: sex, age, region and country of birth, reason for residence; (2) Statistics Norway's Events Database: type of residential area, family situation, educational level, number of annual days with unemployment benefits, number of annual days with sickness absence and disability pension; and (3) Norwegian Patient Register: date and cause of inpatient and specialised outpatient healthcare. All data sources in Sweden and Norway were available for the entire duration of the study (1998–2018), except for the Norwegian Patient Register (available since 2008) and data on specialised outpatient healthcare in Sweden (available since 2001).

### Cases

Persons who died by suicide, coded according to International Classification of Diseases version 10 (ICD-10) codes X60–X84, were identified as cases.

### Refugees and the host population

The respective migration agencies in Sweden and Norway grant residence permits to refugees and primarily identify refugees according to the Geneva Convention definition (UNHCR, 2020). The Swedish Migration Agency also considers refugee status according to the following reasons for residence: ‘in need of protection’ or ‘humanitarian grounds’ (Swedish Migration Agency, 2020). Similarly, the Norwegian Directorate of Immigration grants refugee status to convention refugees, resettlement refugees and refugees with ‘other/unspecified’ reason for residence (Statistics Norway, 2016). In this study, an individual was identified as a refugee if the person had received a residence permit in the respective host country (Sweden or Norway) as a refugee according to the definitions mentioned above. For details, refugees were further categorised according to their region (Africa, Asia, other regions) and country of birth (Eritrea, Somalia, other countries from Africa, Afghanistan, Iran, Iraq, other countries from Asia, former Yugoslavian countries and other countries outside Africa and Asia) as well as their duration of residence (0–5, 6–10, 11–15 and >15 years) in the host country. Individuals born in Sweden or Norway were considered as the host population in the respective host country. All other individuals who were neither refugees nor belonged to the host population were categorised as non-refugee immigrants.

### Covariates

The following factors were considered in the multivariate-adjusted analyses: (A) socio-demographic factors (sex, age, educational level, family situation and type of residential area); (B) LMM factors (unemployment, sickness absence, disability pension); (C) healthcare factors (inpatient or specialised outpatient healthcare due to any mental disorders (ICD-10 codes: F00-F99) and specialised healthcare for deliberate self-harm identified by ICD-10 codes X60–X84 and Y10–34 in Sweden and by a devised coding system in Norway) (Qin and Mehlum, 2020). Different coding systems were used in analogy with local coding practices and recommendations in Sweden and Norway (Runeson *et al*., 2016; Hollander *et al*., 2020; Qin and Mehlum, 2020) to minimise underreporting of deliberate self-harm. Age and sex (matching factors) were measured at the time of suicide/matching, and other socio-demographic covariates were measured at the preceding year. LMM and healthcare factors were measured during the 1- and 3-year period, respectively, prior to the suicide for individual cases and their corresponding controls. The covariates were categorised according to Table 1. Missing values for a covariate were coded as a separate category.

**Table 1.** Descriptive statistics of socio-demographic and labour market marginalisation factors of all individuals aged 18–64 years who died by suicide during 1998–2018 in Sweden and Norway and corresponding sex- and age-matched controls from the general population (*N* = 17 572/351 440 and 9443/188 860 cases/controls in Sweden and Norway, respectively), and descriptive statistics of healthcare factors for cases/controls during 2011–2018 (*n* = 6561/131 220 and 3819/76 380 cases/controls in Sweden and Norway, respectively)

Characteristics	Sweden		Norway	
	Death by suicide *n* (%)	Controls *n* (%)	Death by suicide *n* (%)	Controls *n* (%)
Socio-demographic factors^a^ (1998–2018)				
Sex (matching factor)				
Women	5109 (29.1)	102 180 (29.1)	2715 (28.8)	54 300 (28.8)
Men	12 463 (70.9)	249 260 (70.9)	6728 (71.2)	134 560 (71.2)
Age in years (matching factor)				
18–24	1926 (11.0)	38 520 (11.0)	1339 (14.2)	26 780 (14.2)
25–34	3032 (17.3)	60 640 (17.3)	1993 (21.1)	39 860 (21.1)
35–44	3612 (20.6)	72 240 (20.6)	2099 (22.2)	41 980 (22.2)
45–54	4683 (26.7)	93 660 (26.7)	2228 (23.6)	44 560 (23.6)
55–64	4319 (24.6)	86 380 (24.6)	1784 (18.9)	35 680 (18.9)
Educational level (years)				
Compulsory school (0–9)	4891 (27.8)	68 172 (19.4)	3636 (38.5)	44 126 (23.4)
High school (10–12)	8735 (49.7)	164 698 (46.9)	3824 (40.5)	79 295 (42.0)
College or university (>12)	3779 (21.5)	109 341 (31.1)	1717 (18.2)	51 492 (27.2)
Missing	167 (1.0)	9229 (2.6)	266 (2.8)	13 947 (7.4)
Family situation				
Married	3608 (20.5)	140 651 (40.0)	1934 (20.5)	81 753 (43.3)
Single	13 964 (79.5)	210 789 (60.0)	7509 (79.5)	107 107 (56.7)
Type of residential area				
Big cities^b^	6125 (34.9)	130 172 (37.0)	1237 (13.1)	26 387 (14.0)
Others	11 447 (65.1)	221 268 (63.0)	8206 (86.9)	162 473 (86.0)
Labour market marginalisation factors^a^^,^^c^ (1998–2018)				
Unemployed, 1–180 days	2413 (13.7)	31 194 (8.9)	373 (4.0)	3913 (2.1)
Unemployed, >180 days	778 (4.4)	9766 (2.8)	273 (2.9)	3274 (1.7)
Sickness absence, 1–90 days	1922 (10.9)	24 117 (6.9)	1319 (14.0)	18 929 (10.0)
Sickness absence, >90 days	2490 (14.2)	12 287 (3.5)	577 (6.1)	5117 (2.7)
Disability pension^d^ (yes)	4315 (24.6)	26 687 (7.6)	3711 (39.3)	29 291 (15.5)
Healthcare factors (2011–2018)^e^				
History of psychiatric healthcare use (yes)	3428 (52.2)	9622 (7.3)	2032 (53.2)	5013 (6.6)
History of deliberate self-harm (yes)	931 (14.2)	759 (0.6)	484 (12.7)	213 (0.3)
Migration status (1998–2018)				
Host population^f^	15 253 (86.8)	291 170 (82.9)	8537 (90.4)	152 125 (80.5)
Refugees	517 (2.9)	17 049 (4.9)	104 (1.1)	5217 (2.8)
Non-refugee immigrants	1802 (10.3)	43 221 (12.2)	802 (8.5)	31 518 (16.7)

a All socio-demographic and labour market marginalisation factors were measured by the time of suicide, or the year prior to the year of suicide or matching time.

b Stockholm, Gothenburg and Malmö in Sweden and Oslo in Norway.

c ‘No unemployment’, ‘No sickness absence’ and ‘No disability pension’ categories are not presented.

d Individuals having a disability pension during the year before suicide.

e Only cases and their corresponding controls during 2011–2018 were included to ensure comparability between Sweden and Norway. Inpatient or specialised outpatient healthcare due to any mental disorders (ICD-10 codes F00–F99) in the preceding 3 years of suicide or matching time was considered as ‘History of psychiatric healthcare use’. Inpatient or specialised outpatient healthcare for any deliberate self-harm (identified by ICD-10 codes X60–X84 and Y10–Y34 in Sweden and by a devised coding system in Norway (Qin and Mehlum, 2020)) during the same period was considered as ‘History of deliberate self-harm’. ‘No history psychiatric healthcare use’ and ‘No history of deliberate self-harm’ categories are not presented.

f Swedish-born for Sweden and Norwegian-born for Norway.

### Statistical analyses

Conditional logistic regression models yielding multivariate-adjusted odds ratios (aORs) with 95% confidence intervals (95% CIs) were used to test the associations between refugee status and suicide. Analyses were also stratified by refugees' region/country of birth and by refugees' duration of residence in the host country. Three different analytic models were applied (1) Model 1: adjusting for the matching factors (age, sex); (2) Model 2: additionally, adjusting for other socio-demographic factors and (3) Model 3: additionally, adjusting for LMM covariates. As data from the Norwegian Patient Register were not available for the entire follow-up period of the study (1998–2018), for comparability between Sweden and Norway, healthcare factors were adjusted in separate analytical models where only cases and controls during 2011–2018 (to guarantee 3 years of information for these variables) were considered. These analyses were also stratified by sex (women and men) and age groups (18–24, 25–44 and 45–64 years) and included 6561/131 220 and 3819/76 380 cases/controls in Sweden and Norway, respectively. Although our research questions did not focus on non-refugee immigrants, they were considered a separate category in all regression analyses to preserve the matching effect in each case-control stratum. All analyses were performed using SAS 9.4.

## Results

In both Sweden and Norway, individuals who died by suicide, compared with respective control groups, had fewer years of education (27.8 *v.* 19.4% with compulsory education among cases and controls in Sweden, respectively; 38.5 *v.* 23.4% with compulsory education among cases and controls in Norway, respectively) and were only half as likely to be married (Table 1). Compared to controls, cases in both host countries had a higher proportion of unemployed individuals (18.1 *v.* 11.7% among cases and controls in Sweden, respectively; 6.9 *v.* 3.8% among cases and controls in Norway, respectively). Also, more cases than controls in both countries received sickness absence benefits (25.1 *v.* 10.4% among cases and controls in Sweden, respectively; 20.1 *v.* 12.7% among cases and controls in Norway, respectively). Almost three times more cases had disability pension during the year before suicide than their corresponding controls in both countries (Table 1).

During the 3 years before suicide, almost sevenfold more cases than controls received specialised healthcare for any mental disorder in Sweden and Norway (Table 1). Specialised healthcare use for deliberate self-harm was comparable among cases in both countries (Table 1). However, among controls, this proportion was higher in Sweden than in Norway (0.6 *v.* 0.3%, respectively). Among the cases, 517 (3%) and 104 (1%) individuals were refugees in Sweden and Norway, respectively.

### Suicide in refugees in Sweden and Norway

In the multivariate-adjusted analyses, the odds ratios for death by suicide during 1998–2008 in refugees in Sweden and Norway were 0.5 (95% CI 0.5–0.6) and 0.3 (95% CI 0.3–0.4), compared with the Swedish-born and Norwegian-born individuals, respectively. When stratified by region/country of birth, similar statistically significant lower odds were observed for most refugee groups in both host countries (Tables 2 and 3) except for refugees from Eritrea in Sweden (aOR 1.0, 95% CI 0.7–1.6). Although direct comparisons were not possible, in general, the point estimates of the aORs of suicide in specific refugee groups in Norway were comparatively lower than that in the same refugee group in Sweden, the confidence limits were largely overlapping, showing little or no difference in suicide risk among the country-specific groups between the two host countries (Tables 2 and 3).

**Table 2.** Multivariate odds ratios (ORs) with 95% confidence intervals (CIs) for suicide during 1998–2018 in refugees in Sweden, according to region and country of birth, compared with the Swedish-born

	Number of cases/controls	Model 1^a^ OR (CI)	Model 2^b^ OR (CI)	Model 3^c^ OR (CI)
Swedish-born	15 253/291 170	1	1	1
Refugees	517/17 049	**0.57** (**0.53–0.63)**	**0.59** (**0.54–0.65)**	**0.54** (**0.49–0.59)**
Africa (region)	74/2062	**0.68** (**0.54–0.85)**	**0.55** (**0.44–0.70)**	**0.56** (**0.44–0.71)**
Eritrea	24/352	1.27 (0.84–1.92)	0.95 (0.62–1.44)	1.03 (0.67–1.57)
Somalia	14/793	**0.33** (**0.19–0.56)**	**0.25** (**0.15–0.42)**	**0.25** (**0.15–0.43)**
Other countries from Africa	36/917	0.74 (0.53–1.03)	**0.68** (**0.48–0.95)**	**0.65** (**0.46–0.91)**
Asia (region)	217/8543	**0.48** (**0.42–0.55)**	**0.50** (**0.44–0.58)**	**0.45** (**0.39–0.51)**
Afghanistan	16/406	**0.14** (**0.09–0.23)**	**0.16** (**0.10–0.27)**	**0.19** (**0.11–0.30)**
Iran	92/2051	0.85 (0.69–1.05)	0.87 (0.70–1.07)	**0.70** (**0.57–0.87)**
Iraq	38/2904	**0.25** (**0.18–0.34)**	**0.27** (**0.20–0.37)**	**0.24** (**0.17–0.33)**
Other countries from Asia	71/3182	**0.42** (**0.33–0.53)**	**0.42** (**0.33–0.54)**	**0.39** (**0.31–0.50)**
Other regions	226/6444	**0.67** (**0.58–0.76)**	**0.73** (**0.64–0.83)**	**0.66** (**0.58–0.76)**
Former Yugoslavian countries	123/4235	**0.55** (**0.46–0.66)**	**0.65** (**0.54–0.77)**	**0.57** (**0.48–0.69)**
Other countries	103/2209	0.88 (0.72–1.08)	0.86 (0.71–1.06)	0.82 (0.67–1.01)

ORs with 95% CIs in bold indicate statistically significant associations (*p*-value <0.05).

Non-refugee immigrants were included as a separate category in all the models (data not shown).

a Model 1: adjusted for the matching factors: sex and age.

b Model 2: adjusted for Model 1 and other socio-demographic factors: educational level, family situation and type of residential area.

c Model 3: adjusted for Model 2 and labour market marginalisation factors: unemployment, sickness absence and disability pension.

**Table 3.** Multivariate odds ratios (ORs) with 95% confidence intervals (CIs) for suicide during 1998–2018 in refugees in Norway, according to region and country of birth, compared with the Norwegian-born

	Number of cases/control	Model 1^a^ OR (CI)	Model 2^b^ OR (CI)	Model 3^c^ OR (CI)
Norwegian-born	8537/152 125	1	1	1
Refugees	104/5217	**0.34** (**0.28–0.41)**	**0.38** (**0.31–0.46)**	**0.30** (**0.25–0.37)**
Africa (region)	25/1498	**0.28** (**0.19–0.41)**	**0.27** (**0.18–0.40)**	**0.20** (**0.14–0.30)**
Eritrea	11/345	**0.52** (**0.29–0.95)**	**0.44** (**0.24–0.81)**	**0.33** (**0.18–0.62)**
Somalia	9/698	**0.22** (**0.11–0.42)**	**0.22** (**0.11–0.42)**	**0.16** (**0.08–0.31)**
Other countries from Africa	<9^d^/455	**0.18** (**0.08–0.44)**	**0.19** (**0.08–0.45)**	**0.15** (**0.06–0.38)**
Asia (region)	51/2295	**0.38** (**0.29–0.50)**	**0.43** (**0.33–0.58)**	**0.34** (**0.26–0.45)**
Afghanistan	9/367	**0.42** (**0.22–0.81)**	**0.48** (**0.24–0.93)**	**0.44** (**0.22–0.85)**
Iran	13/326	0.69 (0.39–1.20)	0.72 (0.41–1.25)	**0.53** (**0.30–0.93)**
Iraq	11/781	**0.24** (**0.13–0.44)**	**0.28** (**0.15–0.51)**	**0.21** (**0.12–0.39)**
Other countries from Asia	18/821	**0.37** (**0.23–0.59)**	**0.44** (**0.27–0.70)**	**0.35** (**0.22–0.56)**
Other regions	28/1424	**0.34** (**0.24–0.50)**	**0.42** (**0.29–0.61)**	**0.37** (**0.25–0.54)**
Former Yugoslavian countries	26/1185	**0.38** (**0.26–0.57)**	**0.48** (**0.32–0.71)**	**0.43** (**0.29–0.64)**
Other countries	<9^d^/239	**0.14** (**0.04–0.57)**	**0.15** (**0.04–0.62)**	**0.14** (**0.03–0.55)**

ORs with 95% CIs in bold indicate statistically significant associations (*p*-value <0.05).

Non-refugee immigrants were included as a separate category in all the models (data not shown).

a Model 1: adjusted for the matching factors: sex and age.

b Model 2: adjusted for Model 1 and other socio-demographic factors: educational level, family situation and type of residential area.

c Model 3: adjusted for Model 2 and labour market marginalisation factors: unemployment, sickness absence and disability pension.

d Due to the risk of identification of individuals, if the number of suicides is <9, it is not reported.

### Duration of residence and suicide in refugees

According to their duration of residence in years, the aORs for suicide in refugee groups in Sweden and Norway ranged 0.5–0.6 and 0.3–0.4, respectively, compared with the respective host population. Increasing duration of residence did not seem to affect the aORs for suicide in refugees in Sweden and Norway (Table 4).

**Table 4.** Multivariate odds ratios (ORs) with 95% confidence intervals (CIs) for suicide during 1998–2018 in refugees in Sweden and Norway, stratified by duration of residence in the respective host country, in comparison with the Swedish-born and Norwegian-born population, respectively

Duration of residence (years)	Number of cases/controls	Model 1^a^ OR (CI)	Model 2^b^ OR (CI)	Model 3^c^ OR (CI)
Swedish-born	15 253/291 170	1	1	1
Refugees in Sweden	517/17 049	**0.57** (**0.53–0.63)**	**0.59** (**0.54–0.65)**	**0.54** (**0.49–0.59)**
0–5	72/3175	**0.43** (**0.34–0.54)**	**0.47** (**0.37–0.59)**	**0.48** (**0.38–0.60)**
6–10	94/3571	**0.50** (**0.41–0.61)**	**0.55** (**0.45–0.68)**	**0.52** (**0.42–0.64)**
11–15	129/3829	**0.64** (**0.53–0.76)**	**0.69** (**0.58–0.83)**	**0.60** (**0.51–0.72)**
>15	222/6474	**0.65** (**0.57–0.74)**	**0.68** (**0.60–0.78)**	**0.57** (**0.49–0.65)**
Norwegian-born	8537/152 125	1	1	1
Refugees in Norway	104/5217	**0.34** (**0.28–0.41)**	**0.38** (**0.31–0.46)**	**0.30** (**0.25–0.37)**
0–5	31/1608	**0.33** (**0.23–0.47)**	**0.38** (**0.26–0.54)**	**0.28** (**0.19–0.40)**
6–10	25/1376	**0.31** (**0.21–0.47)**	**0.34** (**0.23–0.51)**	**0.28** (**0.19–0.42)**
11–15	18/1059	**0.29** (**0.18–0.46)**	**0.31** (**0.20–0.50)**	**0.26** (**0.16–0.42)**
>15	30/1174	**0.43** (**0.30–0.62)**	**0.47** (**0.33–0.68)**	**0.39** (**0.27–0.56)**

ORs with 95% CIs in bold indicate statistically significant associations (*p*-value <0.05).

Non-refugee immigrants were included as a separate category in all the models (data not shown).

a Model 1: adjusted for the matching factors: sex and age.

b Model 2: adjusted for Model 1 and other socio-demographic factors: educational level, family situation and type of residential area.

c Model 3: adjusted for Model 2 and labour market marginalisation factors: unemployment, sickness absence and disability pension.

### Factors related to socio-demographics, labour market marginalisation and healthcare use, and suicide risk in refugees

Factors regarding socio-demographics (Model 2), LMM (Model 3) and healthcare use (Model 4) had a marginal effect on the risk estimates (aORs) of suicide in refugee groups in Sweden and Norway during 2011–2018 (Table 5). Stratification by age group revealed that refugees aged 18–24 years in both countries had a similar risk of suicide (aOR 1.0, 95% CI 0.7–1.4 and aOR 0.8, 95% CI 0.5–1.4 in Sweden and Norway, respectively), compared with the same-aged individuals from the respective host country. The risk of suicide in other refugee groups, by sex and age, was similar to that of the respective whole refugee population in Sweden and Norway (Table 5).

**Table 5.** Multivariate odds ratios (ORs) with 95% confidence intervals (CIs) for suicide during 2011–2018 in refugees in Sweden and Norway, stratified by sex and age groups, in comparison with the Swedish-born and Norwegian-born population, respectively, belonging to the same sex and age group

Sex and age group	Cases/controls	Model 1^a^ OR (CI)	Model 2^b^ OR (CI)	Model 3^c^ OR (CI)	Model 4^d^ OR (CI)
Refugees in Sweden	271/9139	**0.54** (**0.48–0.61)**	**0.55** (**0.49–0.62)**	**0.51** (**0.45–0.58)**	**0.57** (**0.50–0.65)**
Women	68/2589	**0.49** (**0.38–0.63)**	**0.43** (**0.34–0.55)**	**0.49** (**0.38–0.63)**	**0.56** (**0.43–0.74)**
Men	203/6550	**0.56** (**0.48–0.65)**	**0.60** (**0.52–0.69)**	**0.52** (**0.45–0.61)**	**0.58** (**0.50–0.68)**
18–24 years	33/774	0.81 (0.57–1.15)	**0.69** (**0.48–0.99)**	0.72 (0.50–1.05)	0.96 (0.66–1.41)
25–44 years	119/4150	**0.50** (**0.41–0.60)**	**0.46** (**0.38–0.55)**	**0.47** (**0.39–0.57)**	**0.55** (**0.45–0.68)**
45–64 years	119/4215	**0.53** (**0.44–0.64)**	**0.58** (**0.48–0.69)**	**0.50** (**0.41–0.61)**	**0.53** (**0.43–0.64)**
Refugees in Norway	65/2889	**0.36** (**0.28–0.47)**	**0.38** (**0.30–0.49)**	**0.30** (**0.23–0.39)**	**0.38** (**0.29–0.49)**
Women	13/612	**0.35** (**0.20–0.61)**	**0.34** (**0.20–0.60)**	**0.27** (**0.16–0.48)**	**0.39** (**0.22–0.72)**
Men	52/2277	**0.37** (**0.28–0.48)**	**0.40** (**0.30–0.53)**	**0.31** (**0.23–0.41)**	**0.37** (**0.28–0.50)**
18–24 years	18/429	0.75 (0.47–1.22)	0.74 (0.46–1.21)	0.68 (0.42–1.11)	0.82 (0.48–1.39)
25–44 years	31/1596	**0.30** (**0.21–0.42)**	**0.30** (**0.21–0.44)**	**0.23** (**0.16–0.34)**	**0.33** (**0.22–0.48)**
45–64 years	16/864	**0.32** (**0.19–0.52)**	**0.37** (**0.22–0.61)**	**0.27** (**0.16–0.45)**	**0.29** (**0.17–0.49)**

ORs with 95% CIs in bold indicate statistically significant associations (*p*-value <0.05).

Non-refugee immigrants were included as a separate category in all the models (data not shown).

a Model 1: adjusted for sex in age group-specific analysis and adjusted for age group for sex-specific analysis.

b Model 2: adjusted for Model 1 covariate and other socio-demographic factors (educational level, family situation and type of residential area).

c Model 3: adjusted for Model 2 covariates and labour market marginalisation factors: unemployment, sickness absence and disability pension.

d Model 4: adjusted for Model 3 covariates and healthcare factors: history of psychiatric healthcare use and history of deliberate self-harm.

## Discussion

### Main findings

Compared with the respective host population in Sweden and Norway, all refugee groups, according to their region/country of birth and duration of residence, had lower odds of death by suicide during 1998–2018, except for refugees from Eritrea in Sweden. Although refugees in Norway had a somewhat lower risk of suicide than refugees in Sweden on the aggregate level, the risk of suicide did not vary much when comparing a specific refugee group in Sweden by their country of birth and the same group in Norway. Factors related to socio-demographics, LMM and healthcare use had a marginal influence on the associations between refugee status and suicide and did not seem to affect the association differently in Sweden and Norway. The associations were not modified by sex but younger refugees aged 18–24 years did not have a statistically significant relative difference in suicide risk than their respective host country peers.

Generally, refugee groups in Sweden and Norway showed a lower suicide risk than the respective host population. Potential explanations for these findings, which may appear counter-intuitive to popular thinking, can be health selection processes in refugees arriving to a host country as well as potential differences between refugees and the host population regarding culture and religion-bound views towards mental ill-health and suicidal behaviour (Amin *et al*., 2021*b*). From register data, we could only follow refugees in the post-resettlement period. Therefore, it is probable that refugees who could overcome the stressors during migration and asylum-seeking period in Sweden and Norway are healthier and more resilient than those who could not make it until resettlement. Moreover, meaning and attitude towards suicidal behaviour can vary across cultures and religions (Spallek *et al*., 2015), and these factors may have beneficially affected the suicide risk in favour of refugees. Both in Sweden and Norway, the majority host population is considered more secular than the immigrant population (Kasselstrand and Mahmoudi, 2020), and refugees in these countries may have a more negative attitude towards suicide which provides a deterrent.

### Comparison with previous studies

We found that aORs for suicide in refugees in Sweden and Norway were 0.5 and 0.3, respectively. These findings are consistent with previous cohort studies where refugees in Sweden (Amin *et al*., 2021*a*) and male refugees in Denmark (Norredam *et al*., 2013) had 0.6 and 0.4 times lower suicide risk, respectively, compared with the respective host populations. Norredam *et al.* also reported lower suicide mortality for male refugees from Iraq and former Yugoslavian countries (rate ratios 0.5 and 0.1, respectively) (Norredam *et al*., 2013) which closely resembles the aORs found in our study for refugees from these countries resettling in Sweden and Norway. Moreover, Amin *et al*. reported similar lower hazard ratios for suicide among refugees from Iraq and former Yugoslavian countries (Amin *et al*., 2021*a*). Although the same study additionally reported risk estimates for suicide among refugees in Sweden migrating from Somalia, Afghanistan and Iran, a comparison was not possible due to lack of statistical power in their analyses.

Our sex-stratified analyses did not reveal any modifying role of sex. However, when stratifying by age groups, refugees aged 18–24 years in Sweden and Norway did not have a statistically significant relative difference in suicide risk than their Swedish-born and Norwegian-born peers; all other refugee groups by age had similar lower risk estimates (aORs) to the risk among refugees altogether. These results partially agree with the findings reported by Amin *et al*. where female refugees aged 16–24 years had a similar risk of suicide compared with their Swedish-born peers of the same sex and age (Amin *et al*., 2021*a*). Previously, Guillot *et al.* showed that the relative risk of mortality among migrants varies by age, and the all-cause mortality advantage was less pronounced among younger migrants (Guillot *et al*., 2018). Their results supported the hypothesis that any positive health selection and cultural effect on migrant mortality advantage are more relevant for older adults. Our results complement these prior findings and may suggest that the suicide mortality advantage seen among older refugees is probably not present to the same extent among young refugees. While tentative, it is not clear why the apparent protectiveness of cultural norms and religious beliefs could be less influential for youth suicidal behaviour than that among older adults. Although highly speculative for this context, some literature suggests that religious beliefs tend to intensify with increasing age for some (Bengtson *et al*., 2015), while older adults may also have more stigmatising attitudes towards suicidal behaviour than youth (Pereira and Cardoso, 2019). Future studies should investigate risk and protective factors for suicidal behaviour among young refugees.

### Risk of suicide for refugees in Sweden and Norway

On the aggregate levels, refugees in Sweden seemed to have somewhat higher suicide mortality than refugees in Norway. Higher psychiatric healthcare use (Abebe *et al*., 2017; Amin *et al*., 2021*b*; Amin *et al*., 2021*c*) and better labour market integration among refugees in Norway than refugees in Sweden (Hernes *et al*., 2019) may have contributed to these small relative differences in suicide risk. However, we may consider this explanation less likely for several reasons. First, our stepwise adjusted models for LMM and healthcare factors did not reveal major differences in changes in estimated suicide risk between Sweden and Norway. Additionally, in the stratified analyses by specific country of birth, we could not see such differences between a specific country of birth group in Sweden and the same group in Norway, even after considering some slightly underpowered sub-analyses and one exception, namely the results concerning refugees from Eritrea. Furthermore, due to lack of data, we could not estimate absolute risk differences for suicide mortality in refugees, limiting the possibility of drawing firm conclusions regarding these differences in aORs for suicide mortality between refugees in Sweden and Norway. For these reasons, we interpret the suicide mortality for refugees in Sweden and Norway to be rather similar. Therefore, our results may suggest that regarding suicide risk, the inherent resilience among specific refugee groups and their attitudes towards suicide could be more protective than other extrinsic environmental and country-level structural factors in the host country.

### Duration of residence and suicide risk

Our results did not support the hypothesis that a longer duration of residence increases the risk of suicide for refugees, and after a certain period of stay in a host country, suicide rates in refugees may converge with the suicide rates in the majority host population. A few previous studies (Nasseri and Moulton, 2011, Hollander *et al*., 2020) reported convergence of suicide rates in immigrants (refugees and non-refugee migrants). Although our results did not conform to those findings, we could disentangle some of the heterogeneity within immigrant groups by focusing only on refugees. A relatively constant risk of suicide over time in refugees in Sweden and Norway also strengthens the alternative hypothesis that suicide risk in this specific group is influenced more by intrinsic cultural, religious and resiliency factors, compared with psychological factors from past trauma or environmental factors experienced in the post-resettlement period.

### Strengths and limitations

The main strength of this study is the case-control study design using registered data of high-quality (Gjertsen, 2002; Ludvigsson *et al*., 2011; Bakken *et al*., 2015; Brooke *et al*., 2017; Bakken *et al*., 2019; Ludvigsson *et al*., 2019) that covered the entire population of Sweden and Norway and thus minimised the risk of selection or recall bias. Another strength is the long follow-up time (21 years). Due to these strengths, we could estimate the risk of a relatively rare outcome measure (suicide) in a minority group like refugees and stratify by some specific region/country of birth. Moreover, we were able to check if the association between refugee status and subsequent suicide was confounded by several factors related to socio-demographics, LMM and healthcare use.

Our results should be interpreted considering some limitations. First, it is not straightforward to compare the ORs for death by suicide in refugee groups in Sweden and Norway because the reference category in each country was the respective majority host population. However, based on data from the World Health Organization, suicide rates in the total population in Sweden and Norway remained fairly stable and comparable between 1998 and 2018 (WHO, 2021), and we assume that these rates were almost similar between the comparison groups, Swedish-born and Norwegian-born, during the follow-up period. Second, case ascertainment procedures are not standardised among different regions/countries and across time. However, national guidelines in Sweden and Norway are quite similar (Gjertsen, 2002; Tøllefsen *et al*., 2015; Brooke *et al*., 2017). Moreover, we did not include deaths due to undetermined intent (ICD-10 codes: Y10–34Y) as suicides because, for such cases, different levels of misclassification may exist between Sweden and Norway (Tøllefsen *et al*., 2015). Third, although we could adjust for an array of covariates as confounders, different measurement practices in Sweden and Norway may have led to differential measurement errors and residual confounding. For example, the regulations for unemployment or sickness absence benefit and disability pension differ between Sweden and Norway, and these factors can be measured differently between the countries (Norwegian Ministry of Labour and Social Affairs, 2020; Swedish Social Insurance Agency, 2020). However, this probably did not bias our results as these factors had a marginal influence on the risk estimates that were similar in both countries. Also, we could not measure and control for the healthcare factors in the analyses, including the entire study population, due to different timelines of the national coverage of the Swedish and Norwegian patient registers. Still, the odds ratio estimates for suicide mortality among refugees in the restricted study population where we could adjust for the healthcare factors fairly resembled the estimates in the analyses for the whole study population without such adjustment. Furthermore, direct comparisons of the suicide rates between refugees' country of birth and the rates in host countries become difficult because data are either lacking or of differential quality due to differences in registration practices among countries. Even then, a comparison among suicide rates in these countries gives some perspectives. Among the most refugee-generating countries, age-standardised suicide rates in Afghanistan, Iran and Iraq (6.0, 5.1 and 4.7 per 100 000 population, respectively) were around half of the rates in Sweden and Norway (10.0 and 12.4, respectively) in 2019 (WHO, 2021). In contrast, the rates in Eritrea and Somalia were much higher (17.3 and 14.7, respectively) (WHO, 2021). Given the variations among these country-specific suicide rates and considering the fact that data were not available regarding religion and attitudes towards suicidal behaviour, we could not confirm if factors related to culture/religion were the primary determinants of lower suicide risk among refugees in Sweden and Norway. To overcome this limitation, information on religiousness and how people view mental disorders and suicidal behaviour is necessary for further investigations. Finally, it is clearly not possible to generalise our findings to refugees living in camps or awaiting an asylum decision, or to refugees who settled in countries with significantly different healthcare and welfare systems or countries that adopt much stricter migration policies than Sweden and Norway.

## Conclusion

Compared with the majority host population in Sweden and Norway, refugees in both host countries had almost similar mortality advantages concerning suicide. Although on the aggregate levels, refugees in Norway seemed to have a mortality advantage regarding the risk of suicide than refugees in Sweden, we could not see such differences between a specific country of birth group in Sweden and the same group in Norway. Also, suicide risk did not vary much across refugee groups by their duration of residence, sex and age except for younger refugees aged 18–24 who did not have a statistically significant relative difference in suicide risk than their respective host country peers. Factors related to socio-demographics, LMM and healthcare use had a marginal influence on the associations between refugee status and suicide in both Sweden and Norway. These results may suggest that other factors relevant to health and resiliency as well as culture/religion-bound attitudes towards suicidal behaviour in refugees could be more influential for their suicide risk after resettlement than other post-migration environmental and structural factors in the host country.

## Data Availability

The data used in this study cannot be made publicly available due to privacy regulations. According to the General Data Protection Regulation, the Swedish law SFS 2018:218, the Swedish Data Protection Act, the Swedish Ethical Review Act, and the Public Access to Information and Secrecy Act, these types of sensitive data can only be made available for specific purposes, including research, that meet the criteria for access to this sort of sensitive and confidential data as determined by a legal review. Readers may contact Professor Kristina Alexanderson (kristina.alexanderson@ki.se) regarding the data in Sweden and Professor Ping Qin (ping.qin@medisin.uio.no) regarding the data in Norway.
